# Clinical and Molecular Analysis in Patients with Peutz-Jeghers Syndrome

**DOI:** 10.5152/tjg.2024.23262

**Published:** 2024-05-01

**Authors:** Pınar Günay Aslan, Ahmet Okay Çağlayan, Elçin Bora, Altuğ Koç, Hilal Yucel, Ayfer Ülgenalp, Yeşim Öztürk, Gül Şeker, Mesut Akarsu

**Affiliations:** 1 Department of Internal Medicine, Dokuz Eylül University School of Medicine, İzmir, Türkiye; 2 Department of Medical Genetics, Dokuz Eylül University School of Medicine, İzmir, Türkiye; 3 Department of Molecular Medicine, Institute of Health Sciences, Dokuz Eylül University, İzmir, Türkiye; 4 Division of Pediatric Gastroenterology, Department of Pediatrics, Dokuz Eylül University School of Medicine, İzmir, Türkiye; 5 Division of Gastroenterology, Department of Internal Medicine, Dokuz Eylül University School of Medicine, İzmir, Türkiye

**Keywords:** Peutz–Jeghers syndrome, next-generation sequencing, multiplex ligation-dependent probe amplification, genotype–phenotype relationship

## Abstract

**Background/Aims:**

Peutz–Jeghers syndrome (PJS) is a rare hereditary disorder linked to increased cancer risk due to specific genetic variants in the *STK11 *gene. This study aimed to assess disease manifestations, genetic profiles, and genotype–phenotype correlations in PJS patients.

**Materials and Methods:**

Twenty patients from 14 families with PJS who were followed up at our clinic between 2011 and 2021 were included. Genetic susceptibility to hereditary cancers was assess–ed using targeted next-generation sequencing (NGS) and multiplex ligation-dependent probe amplification (MLPA) of the* STK11* gene. Clinical data were also collected and analyzed in conjunction with the genetic findings.

**Results:**

Initial symptoms appeared around 18.9 years, predominantly abdominal pain and intussusception. Mucocutaneous lesions were found in 85%, and hamartomatous polyps in 90%. Dysplastic polyps were found in 4 patients, with 3 cases of malignancy. Next-generation sequencing identified 11 pathogenic and 3 likely pathogenic mutations, including 3 novel *STK11 *variants (LRG_319: c.598-8_601del, LRG_319: c.708_718del, and LRG_319: c.146_147del). Next-generation sequencing diagnostic rate was 78.5% (11/14), and the overall diagnostic rate with NGS and MLPA studies was 85.7% (12/14). Patients without *STK11 *mutations had later symptom onset and potentially lower cancer risk. Truncated mutations are associated with earlier symptoms and elevated cancer risk.

**Conclusion:**

This is the first PJS case series in Türkiye using the NGS and MLPA methods. It reports 3 novel mutations and emphasizes the genotype–phenotype relationship of PJS. With further studies, the genotype–phenotype relationship of *STK11* variants will be better understood.

**Main Points**

- Symptoms start at 18.9 years on average, often with abdominal pain and intussusception. Eighty-five percent have mucocutaneous lesions, and 90% have hamartomatous polyps.
- Next-gen sequencing finds 11 pathogenic and 3 likely pathogenic mutations, including 3 novel STK11 variants. Next-generation sequencing diagnoses 78.5%, rising to 85.7% with multiplex ligation-dependent probe amplification.
- *STK11* mutations relate to symptom onset; non-mutated patients have delayed symptoms, and lower cancer risk; truncating mutations show earlier symptoms, and higher polyp/cancer risk.

## Introduction

Peutz–Jeghers syndrome (PJS) is a rare autosomal dominant cancer predisposition syndrome, with an estimated prevalence ranging from 1 in 50 000 to 1 in 280 000.^1-3^ Its hallmark features include hamartomatous polyps within the gastrointestinal tract, particularly the small intestine, and distinctive mucocutaneous pigmentation, often emerging during adolescence.^4^

Diagnosis of PJS relies on clinical presentation and family history, as outlined in Table 1.^5^ This syndrome associated with an elevated risk of gastrointestinal and extragastrointestinal cancers,^6^ with a reported lifetime risk of 55%-85%.^5^ Genetic analysis contributes to diagnosis confirmation, clinical interpretation, and early risk detection.

Peutz–Jeghers syndrome arises from germline mutations in the *STK11* tumor suppressor gene located on chromosome 19p13.3. This gene encodes a serine/threonine-protein kinase that regulates the mammalian rapamycin target pathway.^7^ According to the Human Gene Mutation Database (http://www.hgmd.cf.ac.uk), over 400 pathogenic or likely pathogenic STK11 mutations have been recorded in individuals with PJS.^8^

Despite extensive genotype–phenotype research, correlation between *STK11* pathogenic variants and associated phenotypes remains debated.^9^ Amos et al^10^ reported that individuals with truncating variants had similar onset ages for initial polyps, whereas those with missense variants exhibited later onset. Similarly, Salloch et al^11^ found higher GI surgeries, increased polyp count, and earlier polypectomy age in cases with truncating *STK11* variants.

Conflicting results were reported regarding the influence of variant type and location on cancer risk and larger studies showed no influence of variant type or location on cancer risk.^12-14^

This study mainly aims to investigate the genetic etiology, establish genotype–phenotype correlations, and provide a comprehensive clinical and genetic profile of patients with PJS in a Turkish cohort. Utilizing a targeted next-generation sequencing (NGS) panel and multiplex ligation-dependent probe amplification (MLPA) analysis, the study seeks to contribute insights into the relationship between *STK11* gene variants and the clinical manifestations of PJS.

## Materials and Methods

This study was approved by the Dokuz Eylül University Non-Interventional Research Ethics Committee (No: 2021/20-44, Date: April 19, 2021). The study included patients followed at the Gastroenterology and Pediatric Gastroenterology Clinics at Dokuz Eylül University Faculty of Medicine from January 2011 to April 2021. Patients meeting at least 1 World Health Organization diagnostic criterion for PJS (Table 1) and having completed genetic testing were enrolled in the study, with their medical records reviewed retrospectively. Written informed consent was obtained from the patients who agreed to take part in the study.

Demographic data (age, gender, age at diagnosis), clinical features (initial symptoms, presence of mucocutaneous skin lesions, polyp count and size in stomach, small intestine, and colon), family history, genetic analysis outcomes, and whether patients were diagnosed with cancer during follow-up were recorded. The median follow-up duration was 68.4 months.

### Targeted Next-Generation Sequencing

The DNA was extracted from peripheral venous blood samples using the QIAamp® DNA Blood Mini Kit with the commercial QIAcube (Qiagen, Germany) following the manufacturer’s protocol. Subsequently, the “Hereditary Cancer Susceptibility NGS panel” (Oncorisk Gene Panel) was performed, employing the “Celemics OncoRisk V3” NGS kit (Oncorisk, Celemics, South Korea). This panel encompasses 30 genes relevant to prediagnosed cases and was sequenced on the Nextseq 500 NGS platform (Illumina, CA, USA). Bioinformatic analysis of the obtained data was conducted using the Variant Analysis Platform for Genomics (SEQ analysis platform, Genomize, Türkiye). For each case, the STK11 gene achieved a minimum 94% coverage at a depth of 20× (Supplementary Table 1). Variants present in the ClinVar database classified as Benign or Likely Benign, as well as variants with an allelic frequency exceeding 5% in publicly available datasets like Exome Sequencing Project, 1000 Genomes Project, or Exome Aggregation Consortium, were excluded. Both coding and intronic regions (up to the kit’s coverage) were included in the analysis. Variant pathogenicity was assessed following the 2015 American College of Medical Genetics and Genomics (ACMG) guidelines,^15^ with inputs from the ClinVar database, Varsome (Saphetor, Switzerland), Franklin (Genoox, CA, USA) analysis platforms, and the SEQ platform. All identified variants underwent visual scrutiny using the Integrative Genomic Viewer software.

### Multiplex Ligation-Dependent Probe Amplification Analysis for *STK11*

For patients in whom no mutations were detected using the targeted multigene NGS panel, the SALSA® MLPA® P101 STK11 probe mix (Microbiology Research Centre [MRC]-Holland, Amsterdam, Netherlands) was employed to screen for deletions and duplications within the STK11 gene.

Subsequent analysis of the obtained data was performed using the Coffalyser.Net software provided by MRC Holland. The assessment of values was based on the ratios derived from the analysis.

## Results

### Clinical Characteristics of Patients with Peutz–Jeghers Syndrome

Among the 20 included patients, the mean age was 28.05 years (5-54). Among them, 13 (65%) were female and 7 (35%) were male. The average age at the onset of symptoms was 19.05 years (1-41). The initial hospital visits were prompted by abdominal pain (HP:0002027) in 7 patients (35.0%), intussusception (HP:0002576) in 7 patients (35.0%), and rectal prolapse (HP:0002035) in 2 patients (10.0%). Mucocutaneous skin lesions were observed in 17 patients (85%). Although the lesions typically appeared before the initial complaint, they were not the primary reason for hospital admission.

Out of the participants, 15 (75%) had a family history, while 5 had no such history. Diagnostic criteria were met by 13 patients (65%), 4 patients (20%) fulfilled 2 of the criteria, and 3 patients (15%) met only 1 criterion (Table 1).

Among the 20 patients, 18 underwent endoscopy and colonoscopy using the double-balloon enteroscopy technique. Gastric polyps were observed in 11 patients, small intestine polyps in 17 patients, and colon and rectum polyps in 13 patients (Table 2).

Dysplastic polyps were detected in 4 patients, and 3 patients developed breast, ovarian, and colorectal cancer. While 19 patients are actively being followed in our clinic, 1 patient died in 2019 due to complications related to ovarian cancer.

### Genetic Characteristics of Patients with Peutz–Jeghers Syndrome

**Figure 1.**
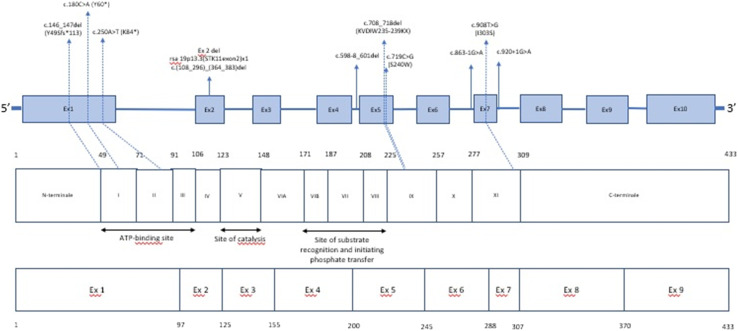
Schematic representation of the *STK11* gene and mutations detected in the study. Modified from^9,10^

Pathogenic or likely pathogenic variants in STK11 were identified in 14 patients using the NGS panel (Table 3, Figure 1). Among these, 3 patients carried novel mutations: LRG_319: c.598-8_601del, LRG_319: c.708_718del p.Asp237Glyfs*26, and LRG_319: c.146_147del (Table 4). In cases where NGS analysis failed to detect pathogenic or likely pathogenic variants (6 patients), del/dup analysis using MLPA was conducted. Notably, exon 2 deletion was identified in 1 patient among these 3 cases. Additionally, a PTEN mutation (LRG_311: c.697C>T p.(Arg233*)) located in exon 7 was detected in a patient, prompting genetic counseling (Table 5).

### Patient with LRG_319: c.598-8_601del

A 17-year-old patient presented with symptoms of nausea and vomiting. Physical examination revealed mucocutaneous lesions on the lower lip and buccal mucosa. The family history was unremarkable. Biopsy and endoscopy revealed the presence of duodenal polyps with hamartomatous features.

### Patient with LRG_319: c.708_718del p.Asp237Glyfs*26

A 25-year-old male patient presented with rectal bleeding. The family history was unremarkable. Physical examination did not reveal any mucocutaneous polyps. Following the report of abdominal pain, the patient underwent endoscopic evaluation, revealing the presence of multiple hamartomatous polyps in the gastric, duodenal, and small intestinal regions.

### Patient with LRG_319: c.146_147del

A 1-year-old male patient was brought to a pediatric outpatient clinic due to rectal prolapse. During the physical examination, a vermillion mucocutaneous lesion was noted. The patient’s family history is notable, as the maternal grandmother had the same condition. Subsequent to complaints of abdominal pain, an endoscopic evaluation revealed multiple hamartomatous polyps in the gastric, duodenal, and small intestinal regions. Follow-up assessments disclosed the presence of multiple rectal polyps, with 2 of them exhibiting dysplasia. As a result, a colorectal resection was performed, involving the removal of a 20 cm segment extending from the colon to the sigmoid colon. Furthermore, an intraluminal mass and intussusception were identified in the distal jejunum. In addition to the STK11 mutation, the patient exhibited MUTYH mutation and CHEK2 variants (Table 5). Notably, the patient’s age at diagnosis was significantly lower than both the study’s mean age of 19 and the mean age reported in the literature. The early onset of symptoms and the implementation of prompt polypectomy could be linked to a truncated mutation. Nonetheless, it’s worth considering that other mutations beyond *STK11 *might contribute to the formation of dysplastic polyps.

## Discussion

This study provides insights into PJS characteristics and genetics. Patient demographics and clinical manifestations align with existing literature, emphasizing the importance of PJS consideration in abdominal symptoms. Notably, mucocutaneous lesions were often overlooked, stressing the need for heightened awareness. Cancer emergence during follow-up confirmed PJS’s malignancy risk. Genetic analysis demonstrated robust detection rates using the NGS panel, while cases with multiple gene alterations highlight the complexity of PJS’s genetic landscape. Pathogenic or likely pathogenic variants were identified in the *STK11* gene, including novel mutations. Our findings emphasize the need for comprehensive genetic analysis and early intervention strategies for effective PJS management.

Our results align with established literature regarding patient demographics, symptom onset, and the prevalence of mucocutaneous lesions.^11,16-18^ These findings reinforce the relevance of PJS as a potential diagnosis in patients presenting with abdominal pain or intussusception. In particular, most patients presented with symptoms other than mucocutaneous lesions, highlighting the need for greater awareness among medical professionals and patients alike.

The distribution of polyps within our cohort mirrors studies from Germany and Taiwan, with significant occurrences in the gastric, small intestinal, and colorectal regions.^11,18^ Importantly, dysplastic polyps were identified in 20% of our patients, reinforcing the necessity of vigilant monitoring due to their inherent malignancy risk.^16,19^

Cancer occurrence during follow-up, while lower in our study, underscores PJS’s malignancy potential,^16,20,21^ and was consistent with previous reports with breast, ovarian, and colorectal cancers emerging in our cohort.^18,22^ This variance might be attributed to the younger age of our patient cohort, suggesting a need for prolonged follow-up to fully assess cancer incidence.

Genetic analysis was conducted using the Hereditary Cancer Susceptibility NGS panel, revealing pathogenic or likely pathogenic changes in PJS-related genes in a substantial portion of patients. The detection rate of 78.5% by NGS panel and 85.7% by NGS panel analysis and MLPA method collectively among patients meeting diagnostic criteria parallels align with previous reviews indicating its efficacy in uncovering pathogenic changes within *STK11* gene.^6^ Our study, in line with previous genetic analyses, reinforces the utility of this approach.^23^

In addition, 3 of our patients (3/20) had alterations in LP/P class genes in more than 1 gene, as detected by panel testing. The occurrence of patients with multiple molecular diagnoses, as observed through whole exome sequencing or clinical exome sequencing methods, has been reported to range between 0.92% and 4% in various studies.^24-29^ The complex etiology arising from mutations in more than 1 gene, leading to similar clinical presentations, adds complexity to the clinical interpretation. Notably, among these 3 cases carrying mutations in more than 1 cancer-related gene, the age of symptom onset is significantly earlier than in the remaining cases. However, the significance of this observation warrants validation through further comprehensive investigations.

Another aim of this study is the exploration of the phenotype–genotype relationship. We compared our cases of cancer and dysplastic polyps with existing literature to explore the phenotype–genotype relationship. In our cohort, 3 patients with mutations developed cancer; 2 with truncated mutations (breast and colorectal carcinoma), and 1 with a missense mutation (ovarian carcinoma). Several studies confirm elevated cancer risk linked to STK11 truncated variants.^30^ Amos et al^10^ reported delayed GI symptoms and later first polypectomy in carriers of missense *STK11 *mutation compared to those with truncations. Also Salloch et al^11^ found that individuals with truncated variants exhibited more polyps, earlier polypectomy, and higher cancer risk. Among patients with truncated mutations, we observed earlier symptom onset and heightened cancer risk, in line with earlier studies.^14,21^ This highlights the potential for tailored follow-up strategies to accommodate varying mutation profiles and optimize patient care carcinoma.

Regarding family patterns, our study aligns with past investigations, demonstrating variability in cancer development among family members sharing the same mutation.^10,12^ Additionally, domain XI mutations correlated with increased dysplasia risk, reinforcing the importance of genetic assessment for tailored patient management.^16^

In conclusion, this study provides valuable insights into the clinical and genetic features of PJS by integrating clinical and genetic insights. The findings underscore the critical importance of of genetic testing in disease management, early diagnosis, vigilant monitoring, and personalized follow-up strategies based on mutation types. Further research is warranted to validate our observations and optimize PJS management strategies.

## Figures and Tables

**Table 1.** Diagnostic Clinical Criteria for Peutz–Jeghers Syndrome^5^

**World Health Organization Criteria 2000**
A: A positive family history of PJS and
1: Any number of histologically confirmed PJS polyps or
2: Characteristic prominent mucocutaneous pigmentation
B: A negative family history of PJS and
1: Three histologically confirmed PJS polyps or
2: Any number of histologically confirmed PJS polyps and characteristic prominent mucocutaneous pigmentation

PJS, Peutz–Jeghers syndrome.

**Table 2.** Demographic and Clinical Characteristics of the Cases

Case & Family No	Age	Gender	Initial Complaint (HPO term and number)	Symptom Onset Age	Mucocutaneous Pigmented Lesion	Family History	# of Criteria Met
1	19	Woman	Abdominal pain HP:0002027	13	On the lip	No	2
2	21	Man	Nausea and vomiting HP:0002017	17	On the lip and buccal mucosa	No	2
3	37	Woman	Anemia HP:0001903	33	Hyperpigmentation in the oral mucosa, macular lesion on the left hand, papular lesion on the right nasal wing	Sibling	3
4	54	Man	Intussusception HP:0002576	41	No	No	1
5	25	Woman	Abdominal pain HP:0002027	20	No	No	1
6	29	Man	Hematochezia HP:0002573	25	No	No	1
7	41	Woman	Abdominal pain HP:0002027	40	On the lip	Son, father, uncle, cousin	3
8	36	Man	Intussusception HP:0002576	9	On the lower lip	Sibling, cousin	3
9.1	21	Man	Intussusception HP:0002576	14	Lip and buccal mucosa	Mother, siblings	3
9.2	19	Woman	Abdominal pain HP:0002027	7	Lip and buccal mucosa	Mother, siblings	3
9.3	43	Woman	Intussusception HP:0002576	20	Lip and buccal mucosa	Children	3
9.4	8	Man	Rectal prolapse HP:0002035	2	Lip and buccal mucosa	Mothers, siblings	3
10	36	Woman	Intussusception HP:0002576	12	Lip and buccal mucosa	Mother, grandfather	3
11	42	Woman	Intussusception HP:0002576	35	On the lip	Son	2
12	13	Man	Rectal prolapse HP:0002035	1	At the edge of the lip	Grandmother	3
13.1	28	Woman	Abdominal pain HP:0002027	22	On the lower lip	Daughter	3
13.2	5	Woman	Intussusception HP:0002576	2	On the lip	Mother	2
14.1	47	Woman	Abdominal pain HP:0002027	40	Lip, intraoral	Daughters	3
14.2	23	Woman	Abdominal pain HP:0002027	17	On the lip	Mother, sister	3
14.3	14	Woman	Constipation HP:0002019	11	Palm	Mother, sister	3

HPO, Human Phenotype Ontology.

**Table 3.** Clinical Features of the Cases

Case and Family No	Gastric Polyp Burden	Maximum Diameter of Gastric Polyp (mm)	Duodenum and Small Intestine Polyp Burden	Maximum Duodenum and Small Intestine Diameter (mm)	Colorectal Polyp Burden	Maximum Colorectal Polyp Diameter (mm)	Malignancy	Survival Status	Mutation Status for *STK11 *Gene	Additional Mutations in Other Genes Included in the Panel
1	1-10	3	1-10	25	1-10	5	No	Alive	Not detected	
2	0	0	1-10	60	0	0	No	Alive	Detected	
3	0	0	1-10	25	1-10	50	Breast invasive ductal carcinoma	Alive	Detected	
4	1-10	4	30-40	60	70-80	5	No	Alive	Not detected	
5	10-20	2	90-100	5	90-100	4	No	Alive	Not detected	*PTEN *
6	1-10	3	20-30	70	1-10	5	No	Alive	Detected	
7	0	0	10-20	30	40-50	40	Metastatic mucinous ovarian cancer	Not Alive	Detected	
8	0	0	20-30	4	1-10	25	Dysplastic Polyp	Alive	Detected	
9.1	1-10	2	10-20	25	0	0	Colorectal Carcinoma	Alive	Detected	
9.2	40-50	2	1-10	5	1-10	6	No	Alive	Detected	*CHEK2*
9.3	1-10	2	1-10	30	1-10	4	Dysplastic Polyp	Alive	Detected	
9.4	1-10	10	0	0	10-20	20	No	Alive	Detected	*CHEK2*
10	60-70	3	40-50	70	10-20	5	No	Alive	Detected	*MUTYH* *CHEK2*
11	Unknown	Unknown	Unknown	Unknown	Unknown	Unknown	No	Alive	Detected	
12	1-10	3	1-10	5	0	0	Dysplastic Polyp	Alive	Detected	
13.1	1-10	4	30-40	40	1-10	4	No	Alive	Detected	
13.2	Unknown	Unknown	Unknown	Unknown	Unknown	Unknown	No	Alive	Detected	
14.1	0	0	1-10	15	1-10	6	No	Alive	Not detected	
14.2	0	0	1-10	2	0	0	No	Alive	Not detected	
14.3	0	0	1-10	3	0	0	No	Alive	Not detected	

**Table 4.** Details of Identified *STK11* Mutations in the Study Population

Case & Family No	HGNC approved gene symbol (OMIM number)	Mutation (LRG_319)	Chromosome and Position (GRCh38)	HGVSc	HGVSp	dbSNP Reference SNP Number	Zygosity	ACMG Classification	Number of P/LP/VUS Variants in the ClinVar Database	Predicted Effect on Protein (REVEL/BayesDel)	Splice Altering Effect (SpliceAI/dbscSNV Ada/RF/varSEAK)	HGMD Database Accession Number
2	*STK11 *(OMIM #602216)	c.598-8_601del	chr19:1220568–1220579	NC_000019.10(NM_000455.5):c.598-8_601del	None (Splice site affected)	Novel	Heterozygos	P	None	X	-/ -/ -/ Class 5	X
3	*STK11 *(OMIM #602216)	c.250A>T	chr19:1207163	NC_000019.10(NM_000455.5):c.250A>T	NC_000019.10(NP_000446.1): p.(Lys84*)	rs137853076	Heterozygos	P	P (6)	-/harmful (Strong) (0.65)	Benign (0)/ -/ -/ Class 1	CM981866
6	*STK11 *(OMIM #602216)	c.708_718del	chr19:1220688–1220698	NC_000019.10(NM_000455.5):c.708_718del	NC_000019.10(NP_000446.1): p.(Asp237Glyfs*25)	Novel	Heterozygos	LP	None	X	-/ -/ -/ Class 2	X
7	*STK11 *(OMIM #602216)	c.719C>G	chr19:1220702	NC_000019.10(NM_000455.5):c.719C>G	NC_000019.10(NP_000446.1): p.(Ser240Trp)	rs730881976	Heterozygos	LP	LP(3), VUS(1)	Harmful (Strong) (0.96)/ harmful (Moderate) (0.48)	X	CM135267
8	*STK11 *(OMIM #602216)	c.908T>G	chr19:1221994	NC_000019.10(NM_000455.5):c.908T>G	NC_000019.10(NP_000446.1): p.(Ile303Ser)	rs727504171	Heterozygos	LP	LP(1)	Harmful (Strong) (0.94)/ Harmful (Moderate) (0.34)	X	X
9.1	*STK11 *(OMIM #602216)	c.180C>A	chr19:1207093	NC_000019.10(NM_000455.5):c.180C>A	NC_000019.10(NP_000446.1): p.(Tyr60*)	rs778376925	Heterozygos	P	P(3)	-/harmful (Strong) (0.65)	Benign (0.01)/ -/ -/ Class 1	CM991149
9.2	*STK11 *(OMIM #602216)	c.180C>A	chr19:1207093	NC_000019.10(NM_000455.5):c.180C>A	NC_000019.10(NP_000446.1): p.(Tyr60*)	rs778376925	Heterozygos	P	P(3)	-/harmful (Strong) (0.65)	Benign (0.01)/ -/ -/ Class 1	CM991149
9.3	*STK11 *(OMIM #602216)	c.180C>A	chr19:1207093	NC_000019.10(NM_000455.5):c.180C>A	NC_000019.10(NP_000446.1): p.(Tyr60*)	rs778376925	Heterozygos	P	P(3)	-/harmful (Strong) (0.65)	Benign (0.01)/ -/ -/ Class 1	CM991149
9.4	*STK11 *(OMIM #602216)	c.180C>A	chr19:1207093	NC_000019.10(NM_000455.5):c.180C>A	NC_000019.10(NP_000446.1): p.(Tyr60*)	rs778376925	Heterozygos	P	P(3)	-/harmful (Strong) (0.65)	Benign (0.01)/ -/ -/ Class 1	CM991149
10	*STK11 *(OMIM #602216)	c.920+1G>A	chr19:1222007	NC_000019.10(NM_000455.5):c.920+1G>A	None (Splice site affected)	rs1131690920	Heterozygos	P	P(2), LP(1)	-/harmful (Strong) (0.65)	Splice-Altering (1)/ Harmful (1)/ Harmful (0.93)/ Class 5	X
11	*STK11 *(OMIM #602216)	rsa19p13.3 STK11(exon2)x1	chr:19p13.3	NC_000019.10(NM_000455.5):c.(108_296)_(364_383) del	X	X	Heterozygos	0.45 – Uncertain (Franklin Genoox)	Not known	X	X	Not known
12	*STK11 *(OMIM #602216)	c.146_147del	chr19:1207059–1207060	NC_000019.10(NM_000455.5):c.146_147del	NC_000019.10(NP_000446.1): p.(Tyr49Serfs*113)	Novel	Heterozygos	P	None	X	Benign (0)/ -/ -/ Class 1	X
13.1	*STK11 *(OMIM #602216)	c.863-1G>A	chr19:1221948	NC_000019.10(NM_000455.5):c.863-1G>A	None (Splice site affected)	rs863224448	Heterozygos	P	LP(1)	-/harmful (Strong) (0.65)	Splice-Altering (1)/ Harmful (1)/ Harmful (0.95)/ Class 5	CS104386
13.2	*STK11 *(OMIM #602216)	c.863-1G>A	chr19:1221948	NC_000019.10(NM_000455.5):c.863-1G>A	None (Splice site affected)	rs863224448	Heterozygos	P	LP(1)	-/harmful (Strong) (0.65)	Splice-Altering (1)/ Harmful (1)/ Harmful (0.95)/ Class 5	CS104386

ACMG, The American College of Medical Genetics and Genomics; dbSNP, The Single Nucleotide Polymorphism Database; GRCh38, Genome Reference Consortium Human Build 38; HGNC, HUGO Gene Nomenclature Committee; HGMD, Human Gene Mutation Database; HGVS, Human Genome Variation Society; HGVSc, the HGVS coding sequence name; HGVSp, the HGVS protein sequence name; Het, heterozygous; Hom, homozygous; LP, likely pathogenic; LRG, locus reference genomic; OMIM, Online Mendelian Inheritance in Man; P, pathogenic; VUS, variant of uncertain significance.

**Table 5.** Cases with Pathogenic/Likely Pathogenic Changes in Genes Other than *STK11*

Case & Family No	HGNC approved gene symbol (OMIM number)	Chromosome and Position (GRCh38)	HGVSc	HGVSp	dbSNP Reference SNP Number	Zygosity	ACMG Classification (P: Pathogenic LP: Likely Pathogenic VUS: Variant of uncertain significance)	Number of P/LP/VUS variants in the ClinVar Database	Predicted Effect on Protein (REVEL/BayesDel)	Splice Altering Effect (SpliceAI/dbscSNV Ada/RF/varSEAK)	HGMD Database Accession Number
5	*PTEN *(OMIM #601728)	chr10:87957915	NC_000010.11(NM_000314.8):c.697C>T	NC_000010.11(NP_000305.3): p.(Arg233*)	rs121909219	Heterozygous	P	P (21)	-/harmful (Strong) (0.56)	Benign (0.01)/ Class 1	CM971277
9.2	*CHEK2 *(OMIM #604373)	chr22:28725099	NC_000022.11(NM_007194.4):c.470T>C	NC_000022.11(NP_009125.1): p.(Ile157Thr)	rs17879961	Heterozygous	LP	P(5), LP(12), VUS(7)	Uncertain (0.54)/ Uncertain (0.11)	-	CM993368
9.4	*CHEK2 *(OMIM #604373)	chr22:28725099	NC_000022.11(NM_007194.4):c.470T>C	NC_000022.11(NP_009125.1): p.(Ile157Thr)	rs17879961	Heterozygous	LP	P(5), LP(12), VUS(7)	Uncertain (0.54)/ Uncertain (0.11)	-	CM993368
10	*MUTYH*(OMIM #604933)	chr1:45332215	NC_000001.11(NM_001048174.2):c.800C>T	NC_000001.11(NP_001041639.1): p.(Pro267Leu)	rs374950566	Heterozygous	P	P(12), LP(3)	Harmful (Strong) (0.95)/ Harmful (Strong) (0.6)	-	CM064129
10	*CHEK2 *(OMIM #604373)	chr22:28694066	NC_000022.11(NM_007194.4):c.1427C>T	NC_000022.11(NP_009125.1): p.(Thr476Met)	rs142763740	Heterozygous	LP	LP(18), VUS(20)	Uncertain (0.45)/ Uncertain (0.1)	-	CM119709

ACMG, The American College of Medical Genetics and Genomics; HGMD, Human Gene Mutation Database; LP, likely pathogenic; P, pathogenic; VUS, variant of uncertain significance.

**Supplementary Table 1.** Next-Generation Sequencing Panel Average Depth and Total Target Region/*STK11* Gene Coverage Rates of Cases

Patient Case&family No	Average Depth	20X Coverage	50X Coverage	*STK11* 20X Gene Coverage	*STK11* 50X Gene Coverage
1	96,48	99.17%	83.72%	100%	72.49%
2	244,8	99.94%	98.65%	100%	93.59%
3	NA	NA	NA	100%	100%
4	241,75	99.99%	98.99%	100%	100%
5	271,76	100%	99.40%	100%	100%
6	193,11	99.85%	97.19%	100%	93.59%
7	270,43	99.96%	99.15%	100%	100%
8	212,52	99.60%	96.72%	100%	100%
9.1	238,15	99.96%	98.85%	100%	99.61%
9.2	414,36	100%	99.89%	100%	100%
9.3	237,49	99.93%	99.09%	100%	100%
9.4	358,44	100%	99.83%	100%	100%
10	166,95	97.92%	99.91%	100%	99.77%
11	116,15	99.49%	91.00%	93.59%	93.59%
12	703,6	100%	100%	100%	100%
13.1	240,19	99.91%	99.22%	100%	100%
13.2	235,28	99.93%	98.57%	100%	100%
14.1	158,97	99.87%	96.36%	100%	88.33%
14.2	280,94	99.98%	99.68%	100%	100%
14.3	87,32	96.90%	71.51%	95.06%	44.13%
